# A novel *CTLA-4* deletion variant in a child with refractory autoimmune hemolytic anemia: molecular and functional characterization

**DOI:** 10.3389/fimmu.2025.1665184

**Published:** 2025-11-19

**Authors:** Feng Chen, Siyu Lei, Caihui Yuan, Zhongjin Xu, Qian Wan, Chongjun Wu, Ting Xiong

**Affiliations:** 1 Department of Hematology, Jiangxi Provincial Children’s Hospital, Nanchang, China; 2 Jiangxi Medical College, Nanchang University, Nanchang, China; 3 Department of Radiology, Jiangxi Provincial Children’s Hospital, Nanchang, China; 4 Department of Endocrine Genetics and Metabolism, Jiangxi Provincial Children’s Hospital, Nanchang, China

**Keywords:** CTLA-4, variant, autoimmune hemolytic anemia, refractory AIHA, children

## Abstract

**Objective:**

As a critical immune checkpoint, cytotoxic T-lymphocyte-associated protein 4(*CTLA-4*)deficiency is a well-established cause of inborn errors of immunity. This study characterizes a novel *CTLA-4* deletion variant identified in a pediatric case of refractory autoimmune hemolytic anemia (AIHA), with the aim of delineating the clinical profile and elucidating the underlying pathogenic mechanism.

**Methods:**

Trio-based whole-exome sequencing (WES) was performed on peripheral blood samples from a 6-year-old female with refractory AIHA and her parents. Candidate variants were validated by Sanger sequencing. Structural modeling of mutant *CTLA-4* was conducted, followed by *in vitro* functional assays in 293T cells to assess mRNA transcription (qPCR) and protein expression (Western blot).

**Results:**

A *CTLA-4* (c.362_391del) variant was identified within the immunoglobulin V-set domain of the *CTLA-4* protein. *In vitro* experiments demonstrated significant reductions in both mRNA and protein expression levels caused by this variant.

**Conclusion:**

The *CTLA-4* (c.362_391del) variant may contribute to refractory AIHA in children. This case highlights the potential necessity of including *CTLA-4* variants in the differential diagnosis of pediatric AIHA, particularly when conventional therapies prove ineffective, and warrants further validation in larger cohorts.

## 1 Introduction

Autoimmune hemolytic anemia (AIHA) is a hematologic disorder characterized by autoantibody-mediated destruction of red blood cells (RBCs) (1). It manifests with a spectrum of clinical features ranging from mild fatigue to life-threatening hemolytic crises. Common presenting symptoms include anemia, jaundice, and hemoglobinuria, while laboratory confirmation relies on the detection of hemolytic autoantibodies via a positive direct antiglobulin test (DAT). Although traditionally considered a rare manifestation of immune dysregulation, emerging evidence suggests associations between AIHA and specific immunodeficiencies (2). In particular, germline heterozygous mutations in the immune checkpoint gene cytotoxic T-lymphocyte-associated protein 4(*CTLA-4*)are increasingly recognized as a cause of refractory autoimmune cytopenias in the pediatric population (3–8). Substantial evidence has established *CTLA-4* gene variants as critical drivers of AIHA pathogenesis (9, 10). Epidemiological studies indicate that approximately 28% of *CTLA-4* variant carriers develop this hematologic complication (11). Functioning as a key immune checkpoint molecule expressed on regulatory T cells (Tregs), *CTLA-4* maintains immune homeostasis by competitively binding CD80/CD86 costimulatory ligands on antigen-presenting cells. This interaction prevents CD28-mediated T-cell activation, thereby serving as a critical brake on adaptive immune responses. Disruption of *CTLA-4*-mediated inhibitory signaling consequently leads to uncontrolled lymphocyte proliferation and multi-organ autoimmunity (12–14).

Patients harboring *CTLA-4* variants frequently exhibit hematologic abnormalities alongside heterogeneous systemic manifestations (14–16). While most reported cases present with initial symptoms during childhood or adolescence (typically before age 18), significant interindividual variability in disease onset and clinical features often complicates timely diagnosis (16–18). We report a pediatric case of refractory AIHA, in whom whole-exome sequencing of a familial trio revealed a novel heterozygous c.362_391del variant in *CTLA-4*. To further clarify the impact of this variant on the disease, identify specific therapeutic targets, establish a definitive diagnosis and provide genetic counseling, and better define the genotype/phenotype correlation, we have characterized this variant to assess its potential role in the disease pathogenesis.

## 2 Materials and methods

### 2.1 Patient

The patient, a 6-year-old girl, was first admitted to our department due to pale complexion. Examinations upon admission revealed severe anemia (Hb: 48 g/L), a marked increase in reticulocytes, jaundice (total bilirubin 44.63 µmol/L, indirect bilirubin 25.32 µmol/L), and dark yellow urine, suggesting hemolytic anemia. Further tests showed a positive DAT (IgG 3+, C3d 2+), normal glucose-6-phosphate dehydrogenase activity, normal levels of folic acid (6.07 ng/mL) and vitamin B12 (519.95 pg/mL), and bone marrow cytology indicative of hyperplastic anemia. The ANA profile and antinuclear antibody test were negative. A definitive diagnosis of AIHA was established.

The patient was treated with methylprednisolone (10 mg/kg/day for 14 days), dexamethasone (0.6 mg/kg/day for 14 days), and intravenous immunoglobulin(IVIG) 2 g/kg, in addition to anti-infective therapy and blood transfusions based on her clinical condition. Despite these interventions, the child continued to pass dark yellow urine, and her hemoglobin level remained low at approximately 68 g/L. In view of this, the case was considered refractory AIHA.

### 2.2 Methods

#### 2.2.1 Trio whole-exome sequencing

Blood samples were obtained from the proband and parents. Genomic DNA was extracted from peripheral blood using DNeasy Blood & Tissue Kit (Qiagen). The samples were used to construct the genomic DNA library and then sequenced on Illumina NovaSeq 6000, with an average cover depth of 90×. The proportion of sequences with a sequencing depth greater than 20× is approximately 98%. The sequencing raw fastq data were aligned to the human reference genome (GRCh37/hg19) using BWA software. The variants were annotated with the Variant Effect Predictor software. Subsequently, the variants were evaluated by ClinVar, OMIM, HGMD and gnomAD databases. Candidate pathogenic variants associated with the clinical phenotype of the proband were verified by Sanger sequencing and were classified according to the ACMG guidelines.

#### 2.2.2 Structural modeling of *CTLA-4* variant

The amino acid sequence of wildtype human *CTLA-4* protein (Uniprot accession: P16410) was obtained from Uniprot database (https://www.uniprot.org/). Its three-dimensional structure was predicted using AlphaFold, with the experimental structure (PDB: 1AH1) serving as a template. The structural quality was validated using PROCHECK, and the model was visualized with UCSF Chimera X.

#### 2.2.3 Functional characterization of *CTLA-4* variant

Human embryonic kidney (HEK) 293T cells were cultured in Dulbecco’s Modified Eagle Medium (DMEM) supplemented with 10% fetal bovine serum. 7.0 × 10^5^ cells per well were seeded in 6-well plates. When the cell confluence reached 80%, the wildtype and mutant eukaryotic recombinant expression vectors p6xHis-*CTLA*-4-wt/mut (1 μg/well) were transiently transfected into 293T cells using Lipofectamine 2000 following the manufacturer’s protocol, and cell samples were harvested 24 hours post-transfection for further experiments.

### 2.3 RNA extraction

Total RNA was extracted from adherent cells using RNA-easy Isolation Reagent (Vazyme, #R701). After removing the culture medium and washing with PBS, cells were lysed directly by adding the reagent and detached via pipetting. The lysate was homogenized and mixed with RNase-free water. Following incubation and centrifugation, the upper aqueous phase was collected. RNA was precipitated with isopropanol, pelleted by centrifugation, and washed twice with 75% ethanol. The pellet was air-dried and dissolved in RNase-free water. RNA concentration and integrity were assessed spectrophotometrically and by agarose gel electrophoresis, respectively. Samples were stored at -80 °C.

### 2.4 qPCR

cDNA was synthesized with HiScript II qRT SuperMix II (Vazyme). qPCR was performed with SYBR Green Master Mix (Vazyme) on a QuantStudio 1 system. Primer sequences were as follows:

CTLA4-F: 5’- GACAAGCTTATGGCTTGCCT -3’; CTLA4-R: 5’- CCACGTGCATTGCTTTGCAG -3’
GAPDH-F: 5’- ACAACTTTGGTATCGTGGAAGG -3’; GAPDH-R: 5’- GCCATCACGCCACAGTTTC -3’

Relative mRNA expression was normalized to GAPDH using the 2^–ΔΔCt method.

### 2.5 Western blot

Harvested cells were washed with cold PBS, and lysed in RIPA buffer (50 mM Tris-HCl, 150 mM NaCl, 1% NP-40, 0.5% sodium deoxycholate, 0.1% SDS) containing protease inhibitors. Lysates were incubated on ice for 30 min and centrifuged at 14,000 × g for 5 min at 4 °C. Protein concentration was determined by BCA assay, and equal amounts of protein were loaded per lane, separated by SDS-PAGE, and transferred to PVDF membranes. After blocking with 5% milk, membranes were incubated overnight with primary antibodies GAPDH (1: 1000, Beyotime, Shanghai, China) and His Tag (1: 1500, Beyotime, Shanghai, China), respectively. After that, membranes were washed three times for 10 min each with TBST and incubated with HRP-conjugated Goat Anti-Mouse IgG (H+L) secondary antibodies (1:1000, Beyotime, Shanghai, China) for 1h at room temperature. Signals were detected using ECL and quantified with ImageJ and normalized to GAPDH.

## 3 Results

### 3.1 Therapeutic management and clinical course

Following the initial diagnosis of refractory AIHA, the patient was treated with rituximab (375 mg/m²); however, the therapeutic response was inadequate. Partial improvement in hemolytic symptoms was achieved after plasma exchange, but these symptoms recurred shortly after remission. Sirolimus was subsequently introduced into the treatment regimen, which led to stabilization of the hemolytic manifestations. During subsequent maintenance therapy with oral prednisone and sirolimus (approximately 2 months of sirolimus treatment), the child developed neurological symptoms, including impaired consciousness that progressed to coma and convulsions.

**Figure 1:**
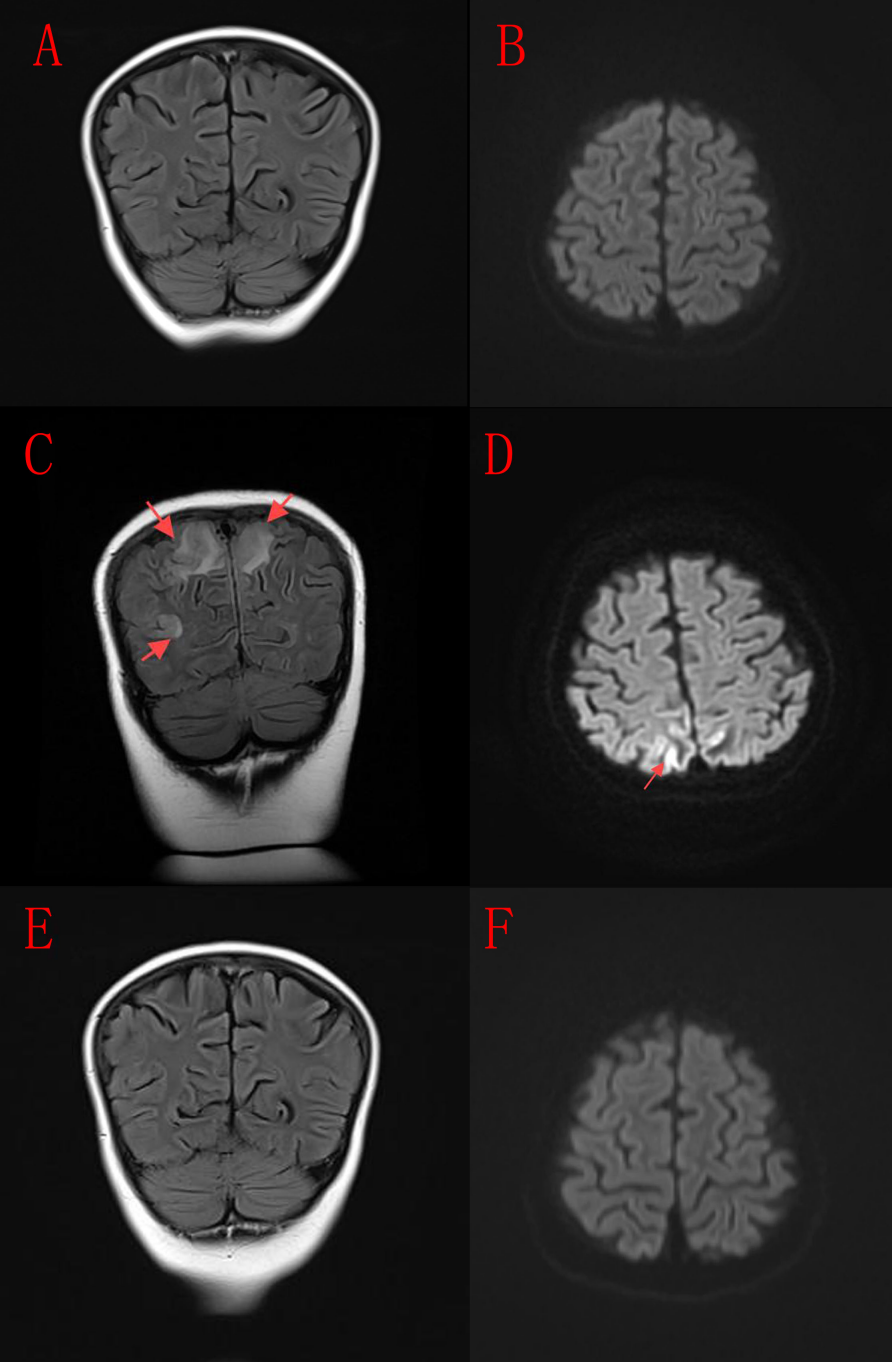
Neuroimaging findings of the patient. **(A, B)** Initial cranial MRI scan obtained prior to the onset of neurological symptoms. **(C, D)** Follow-up cranial MRI after the patient developed neurological symptoms, including convulsions, impaired consciousness, and coma, revealing hyperintense signals on FLAIR and DWI sequences in the bilateral occipital and parietal lobes. **(E, F)** Cranial MRI following antihypertensive therapy, demonstrating near-complete resolution of the abnormal signals.

Emergency hospitalization led to a diagnosis of hypertensive encephalopathy (blood pressure at admission: 147/106 mmHg; cerebrospinal fluid study was normal). Antihypertensive treatment was promptly initiated, resulting in normalization of blood pressure and subsequent imaging showing resolution of the lesions (**Figure 1**), along with clinical improvement. The patient was subsequently discharged and currently remains on oral sirolimus therapy. Follow-up to date has confirmed sustained control of hemolytic activity.

A detailed medical history review revealed a prior diagnosis of immune thrombocytopenia(ITP)in this child. Notably, platelet counts remained within the normal range throughout the current disease course, and no family history of related disorders was documented.

### 3.2 Flow cytometry findings

Immunophenotypic analysis revealed significant dysregulation of lymphocyte subsets prior to therapy, characterized by elevated B-cell counts, expanded CD8+ T-cell populations, an inverted CD4+/CD8+ ratio, and a severe reduction in NK cells. Following the initiation of sirolimus treatment, these parameters showed marked improvement: the CD4+/CD8+ ratio normalized, NK-cell percentages returned to the lower end of the normal range (though absolute counts remained below normal), and CD4+ T-cell percentages and absolute counts were restored to normal levels (**Table 1**).

**Table 1:** Lymphocyte subset dynamics pre-post-sirolimus.

Parameter	Reference range	Post GC/IVIG, pre rituximab/sirolimus	Post rituximab/sirolimus
Percentage values (%)			
Total T lymphocytes (CD3+)	59.5~75.56	63.57	91.7
CD8+ T lymphocytes	19.68~34.06	36.26	37.72
CD4+ T lymphocytes	26.17~41.07	23.53	48.49
Double-positive T lymphocytes	0.15~0.71	0.35	0.43
NK cells	4~26	1.48	5.49
B lymphocytes	10~31	34.65	2.32
Absolute counts (cells/μL)			
Total T lymphocytes(CD3+)	1424~2847	4170.94	2061.19
CD8+ T lymphocytes	518~1127	2378.92	847.8
CD4+ T lymphocytes	686~1592	1544.16	1089.93
Double-positive T lymphocytes	4~25	22.79	9.6
NK cells	227~727	96.87	123.47
B lymphocytes	280~777	2273.5	52.13
Ratio			
CD4+/CD8+ ratio	0.98~1.94	0.65	1.29

### 3.3 Genetic results of the patient

The proband harbors an in-frame deletion variant, c.362_391del, in the *CTLA-4* gene (NM_005214.5). Sanger sequencing confirmed its maternal inheritance (**Supplementary material 1**). This variant is absent from published literature and unreported in the large population database gnomAD. Based on available evidence, it is classified as uncertain significance (VUS).

### 3.4 Structural consequences of the *CTLA-4* variant

**Figure 2:**
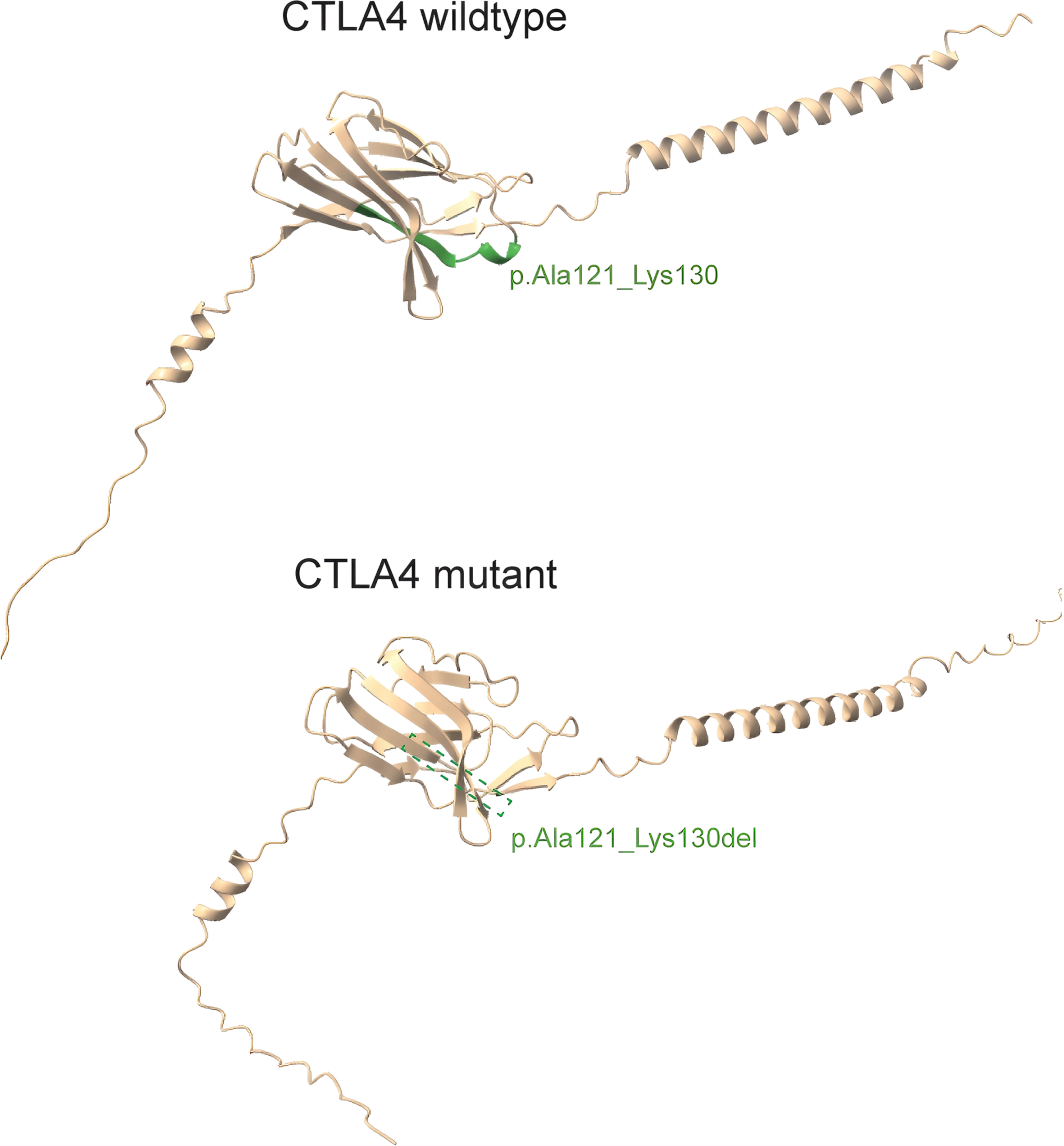
3D protein structures of wildtype *CTLA-4* and *CTLA-4* variant. The upper panel depicted wild-type CTLA4 with the amino acid residues labeled at position p.Ala121_Lys130; the lower panel illustrated that the variant c.362_391 del caused the deletion of residues at position 121 to 130.

The wild-type *CTLA-4* protein (upper panel) exhibits the canonical amino acid sequence, including the region encompassing residues Ala121 to Lys130. In contrast, the mutant *CTLA-4* protein (lower panel) harbors the p.Ala121_Lys130del variant, resulting in the deletion of amino acids 121 to 130 (**Figure 2**).

### 3.5 Functional validation of *CTLA-4* expression

**Figure 3:**
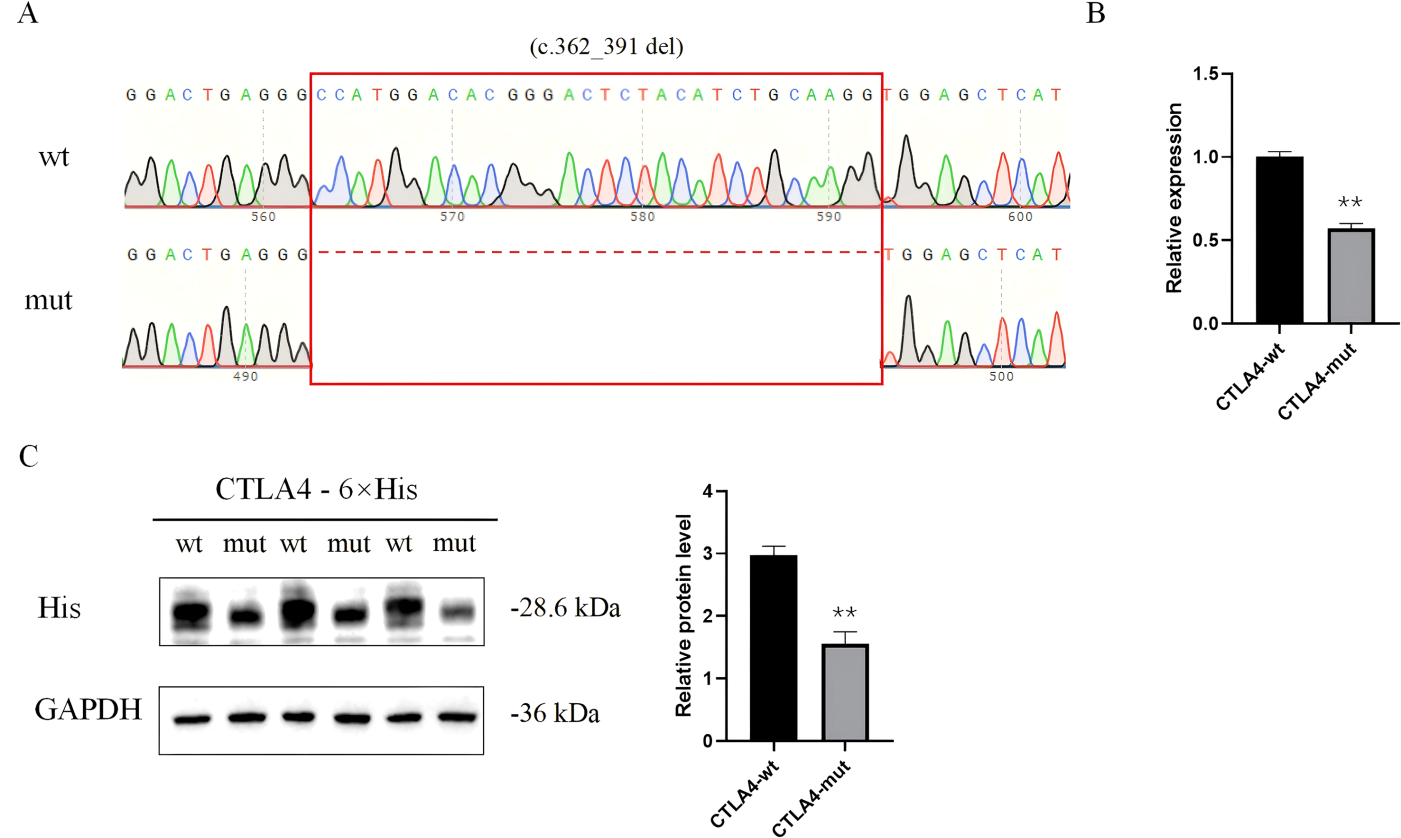
Functional validation of *CTLA-4* expression. **(A)** Sequencing chromatogram demonstrating the successful construction of the mutant vector with the deletion mutation (c.362_391del). **(B)** qPCR showed that the mRNA level of *CTLA-4* was significantly decreased in *CTLA-4*-mut. **(C)** Western blot showed that *CTLA-4*-mut inhibited the protein level of *CTLA-4*.

qPCR analysis revealed a significant reduction in *CTLA-4* mRNA expression in cells expressing the mutant variant compared to the wild-type construct. Corroborating the qPCR results, Western blot analysis demonstrated a marked decrease in mutant *CTLA-4* protein expression relative to wild-type *CTLA-4* (**Figure 3**, **Supplementary Material 2**, **3**).

## 4 Discussion

Standard management of AIHA involves first-line glucocorticoid therapy, with or without the anti-CD20 monoclonal antibody rituximab, complemented by supportive measures such as red blood cell transfusion, erythropoiesis-stimulating agents, IVIG, and plasma exchange for autoantibody removal (19). In this pediatric case, the presentation of refractory AIHA alongside a history of ITP raised suspicion of an underlying immune regulatory disorder. Trio-WES identified a maternally inherited heterozygous *CTLA-4* variant (c.362_391del), classified as VUS. Although *CTLA-4* deficiency, first linked to autoimmune lymphoproliferative syndrome (ALPS)-like dysregulation in 2014 (10, 11), is predominantly diagnosed in adults, the majority of reported cases manifest initial symptoms before age 18. This phenotypic variability contributes to diagnostic complexity and delay (6). This case underscores the critical role of genetic testing in treatment-refractory presentations, as the diagnosis was established only after conventional therapies failed. The patient’s early-onset AIHA (diagnosed at age 6) following resolved ITP highlights the importance of considering inherited immune dysregulation in children with recurrent autoimmune cytopenias, even in the absence of a familial history.

*CTLA-4* deficiency arises from heterozygous loss-of-function variants in the *CTLA-4* gene located on chromosome 2q33.2. This genetic defect underlies a complex phenotype characterized by immune dysregulation, lymphoproliferation, and incomplete penetrance (20). The clinical spectrum encompasses multiorgan autoimmunity and immune-mediated cytopenias due to immune dysregulation (21). Notably, aberrant lymphocyte subsets are hallmark features of *CTLA-4* deficiency. In this pediatric case, pretreatment immunophenotyping demonstrated marked B-lymphocyte expansion concurrent with CD4+ T-lymphocytopenia and an inverted CD4+/CD8+ ratio. The clinical presentation, characterized by AIHA and a history of thrombocytopenia, aligned with established features of the disorder. Following sirolimus initiation, substantial normalization of lymphocyte subsets was observed, evidenced by restoration of the CD4+/CD8+ ratio-a finding that corroborates the diagnosis and confirms therapeutic efficacy. Given the concordance between the clinical presentation, immunophenotypic abnormalities, and the identified genetic variant, we propose that this specific variant is likely associated with the pathogenesis.

Although our investigation of the family history identified no symptomatic carriers among the proband’s mother or other relatives, this finding aligns with the established principle of incomplete penetrance in *CTLA-4* deficiency. Clinical manifestations among variant carriers exhibit high heterogeneity, with an estimated penetrance of 67-71% (15, 16). Consistent with this, a cohort study documented that 8 of 19 adult *CTLA-4* variant carriers remained asymptomatic (11). Notably, reduced *CTLA-4* protein expression in Tregs was observed in both symptomatic patients and asymptomatic carriers, indicating that the variant exerts a functional impact irrespective of clinical phenotype. In this pediatric case, the presentation of refractory AIHA alongside a history of ITP fulfills the clinical criteria for an ALPS-like phenotype. It is important to note that while the clinical presentation is ALPS-like, the genetic etiology in this case is *CTLA-4* haploinsufficiency, which is classified distinctively from classical ALPS (most commonly caused by mutations in the FAS pathway) as an inborn error of immunity with immune dysregulation (17, 21). We acknowledge several limitations in our diagnostic workup. Key biomarkers for classical ALPS, such as the level of double-negative T (DNT) cells (TCRαβ+ CD4- CD8-), were not assessed at the time of initial diagnosis and prior to any treatment. The measurement in future evaluations of similar patients would be invaluable for a more comprehensive phenotyping and differential diagnosis.

Reports on the phenotypic manifestations in pediatric patients harboring *CTLA-4* variants remain limited in the literature (5). In the present case, symptom stabilization was achieved only following the initiation of sirolimus therapy. Current evidence suggests that therapeutic options for symptomatic *CTLA-4* variants include IVIG infusion, corticosteroid therapy, sirolimus, and abatacept (6). While abatacept has demonstrated efficacy in ameliorating autoimmune manifestations, its long-term impact on bone marrow function warrants further investigation (22). Notably, a case of life-threatening refractory AIHA responded to abatacept-based therapy. However, abatacept monotherapy proved insufficient to sustain hemoglobin levels over the long term. A durable therapeutic effect was ultimately achieved with a combination of azathioprine and abatacept (19). In severe cases, abatacept may serve as a bridge to hematopoietic stem cell transplantation (HSCT). For patients ineligible for HSCT, abatacept may be considered a primary treatment option (5). Tsifilis et al. evaluated HSCT outcomes in 40 patients with *CTLA-4* deficiency, reporting a 3-year overall survival rate of 76.7% and a disease-free survival rate of 74.4% (23).

When contextualized within the growing literature on pediatric *CTLA-4* insufficiency, our case shares several hallmark features (3–8). Similar to the cohorts described by Schwab et al. (15), our patient presented in childhood with refractory AIHA, which responded to sirolimus-a therapy increasingly used in this setting. The immunophenotypic abnormalities, particularly B-cell expansion and T-cell subset dysregulation, are also consistent with previous reports (15, 21). Our case adds to this spectrum by describing a novel in-frame deletion within the immunoglobulin V-set domain, a region critical for ligand binding. Furthermore, the severe neurological presentation, though primarily attributed to hypertension, underscores the complex multi-organ involvement that can occur and aligns with other reports of neurological complications in *CTLA-4* deficiency (24, 25), emphasizing the phenotypic variability even in pediatric patients.

Several studies have documented a spectrum of neurological manifestations in patients with *CTLA-4* variants. Characteristic MRI findings in these cases often include diffuse white matter and basal ganglia FLAIR hyperintensities, cerebellar atrophy (24), as well as periventricular, juxtacortical, and cerebellar inflammatory lesions, sometimes with spinal cord involvement (25). In the present case, the child developed significant neurological symptoms-including seizures, altered consciousness, and coma-during concomitant treatment with prednisone and sirolimus. MRI at that time (**Figures 1C, D**) revealed FLAIR and DWI hyperintensities in the bilateral occipital and parietal lobes. The initial MRI performed during the early treatment phase, when the child was free of neurological symptoms, showed no significant abnormalities (**Figures 1A, B**). At the onset of neurological symptoms, the child exhibited markedly elevated blood pressure, and cerebrospinal fluid analysis at that time revealed no notable abnormalities. The imaging findings obtained during the symptomatic phase (**Figures 1C, D**) were consistent with hypertensive encephalopathy. Following timely antihypertensive intervention, both the clinical symptoms and the imaging abnormalities showed significant resolution (**Figures 1E, F**). Although the temporal association between hypertension and symptom onset-coupled with rapid resolution following blood pressure control-strongly supports hypertensive encephalopathy as the primary diagnosis, a contribution from the underlying *CTLA-4* variants cannot be entirely excluded. Notably, the neurological deterioration occurred in the context of treatment with prednisone and sirolimus, both known to elevate blood pressure, while their immunosuppressive effects may have simultaneously masked subinflammatory processes related to the genetic defect. Thus, while hypertensive encephalopathy appears to be the most direct cause of the acute neurological event, the presence of *CTLA-4* variants may have conferred a predisposing background. This case underscores the need for comprehensive clinical vigilance in patients with *CTLA-4* variants, emphasizing consideration of both metabolic and immune-mediated mechanisms in the evaluation of neurological complications.

We conducted a three-dimensional structural visualization analysis of both wildtype and mutant *CTLA-4* proteins. The results revealed a p.Ala121_Lys130del variant, characterized by the deletion of amino acids from positions 121 to 130. This mutated region is situated within the Immunoglobulin V-set domain of the *CTLA-4* protein (amino acids 42-151). This alteration may compromise the protein’s function and stability, thereby significantly impacting the immune response. *In vitro* experiments confirmed successful overexpression of eukaryotic expression vectors p6xHis-*CTLA-4*-wt and p6xHis-*CTLA-4*-mut in 293T cells. qPCR demonstrated that the c.362_391del variant significantly reduced *CTLA-4* mRNA expression. Consistently, Western blotting showed markedly diminished protein levels.

As a single-case study, the generalizability of our findings is inherently limited and warrants confirmation in larger patient cohorts. We recognize that the employed *in vitro* overexpression system may not fully recapitulate the regulatory complexity of primary T cells. Furthermore, the precise mechanism responsible for the reduced mRNA levels (e.g., nonsense-mediated decay) remains to be experimentally verified. Although our data demonstrate a clear loss-of-expression phenotype, a more comprehensive functional interrogation-such as flow cytometric analysis of *CTLA-4* expression on patient-derived Tregs, Treg suppression assays, or direct assessment of ligand binding affinity-was not feasible due to limitations in primary cell availability. We consider these critical analyses a priority for future studies aimed at definitively elucidating the pathogenic mechanism of this novel variant.

## 5 Conclusion

This single-case study expands the recognition of clinical heterogeneity linked to *CTLA-4* variants in pediatric populations, and suggests the potential utility of genetic testing in cases with atypical autoimmune presentations. Our findings in this patient establish the importance of considering *CTLA-4* variants in the differential diagnosis of pediatric AIHA, especially when conventional therapies fail. Early molecular evaluation is recommended to facilitate timely diagnosis and initiate molecular-targeted therapeutic interventions.

## Data Availability

The original contributions presented in the study are included in the article/**Supplementary Material**. Further inquiries can be directed to the corresponding authors.

## Supplementary material

Supplementary FIGURE 1 Genetic results of the patient.

Supplementary FIGURE 2 Anti-GAPDH-*CTLA4*-wt-*CTLA4*-mut.

Supplementary FIGURE 3 Anti-His-*CTLA4*-wt-*CTLA4*-mut.

Supplementary FIGURE 4 Schematic figure.
