# Supplementary figures and images for: A novel CTLA-4 deletion variant in a child with refractory autoimmune hemolytic anemia: molecular and functional characterization
Source: Front Immunol. 2025 Nov 19;16:1665184. doi: 10.3389/fimmu.2025.1665184 (PMC12672875; doi:10.3389/fimmu.2025.1665184)

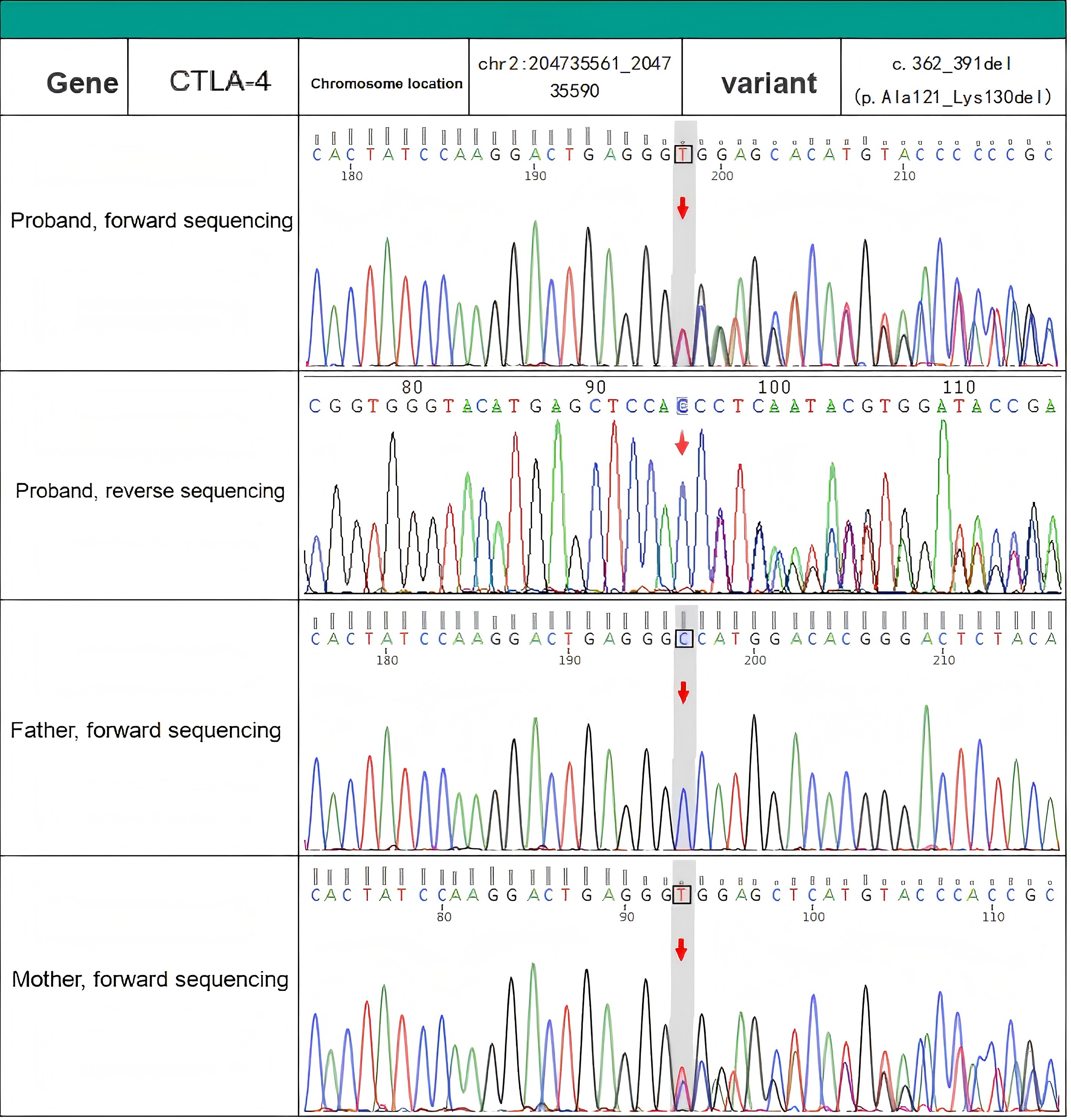

Supplement: Supplementary FIGURE 1 — Genetic results of the patient. [file Image1.jpeg]

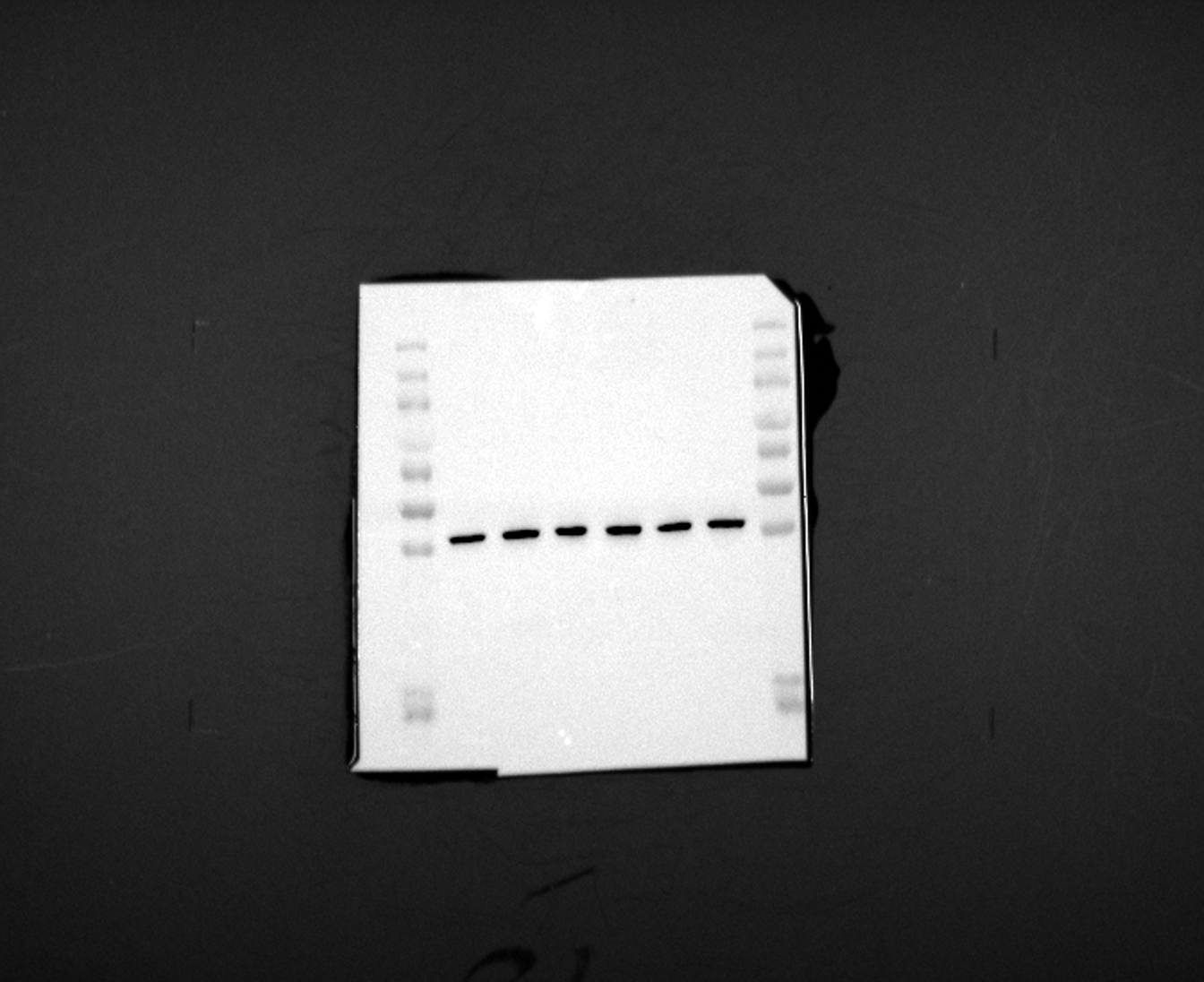

Supplement: Supplementary FIGURE 2 — Anti-GAPDH-CTLA4-wt-CTLA4-mut. [file Image2.tif]

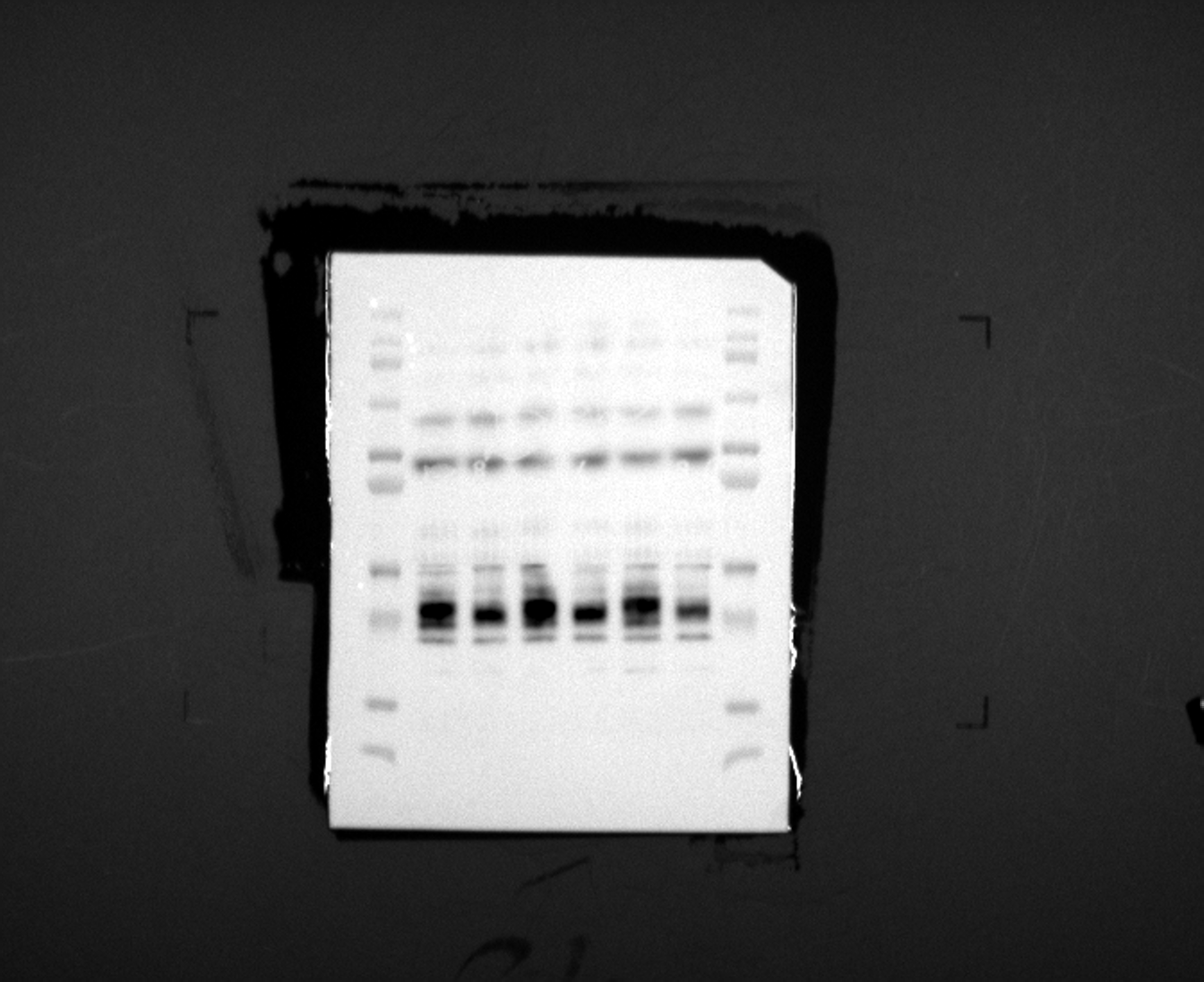

Supplement: Supplementary FIGURE 3 — Anti-His-CTLA4-wt-CTLA4-mut. [file Image3.tif]

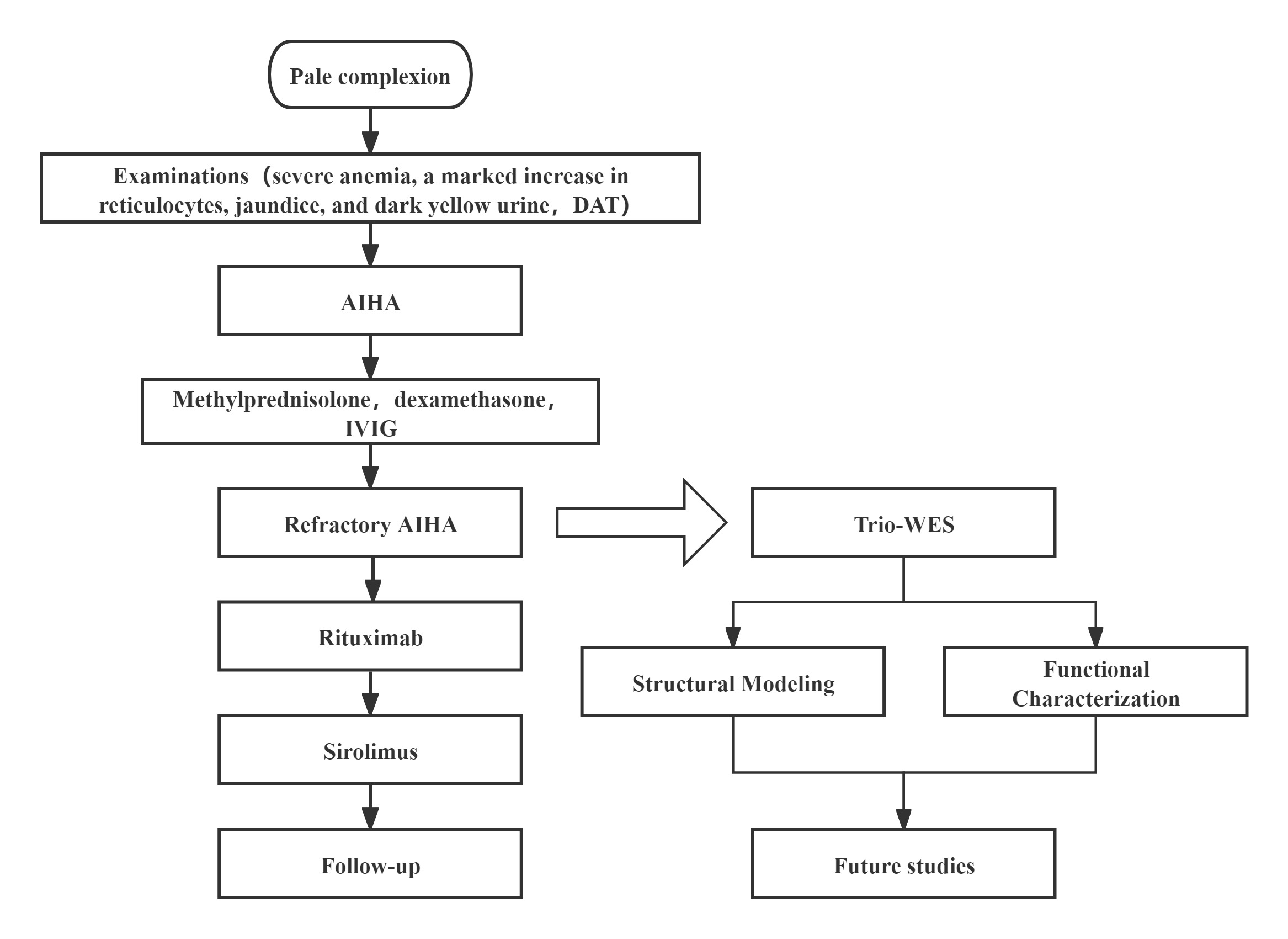

Supplement: Supplementary FIGURE 4 — Schematic figure. [file Image4.jpeg]
